# Polymerized porin as a novel delivery platform for coronavirus vaccine

**DOI:** 10.1186/s12951-022-01469-8

**Published:** 2022-06-07

**Authors:** Zhongqian Yang, Liangqun Hua, Mengli Yang, Weiran Li, Zhaoling Ren, Xiao Zheng, Haoqian Chen, Qiong Long, Hongmei Bai, Weiwei Huang, Yanbing Ma

**Affiliations:** 1 Laboratory of Molecular Immunology, Institute of Medical Biology, Chinese Academy of Medical Sciences and Peking Union Medical College, Kunming, 650031 China; 2 Yunnan University, Kunming, 650091 China; 3 National Kunming High-Level Biosafety Primate Research Center, Institute of Medical Biology, Chinese Academy of Medical Sciences and Peking Union Medical College, Kunming, 650031 China; 4 The Second Affiliated Hospital of Kunming Medical University, Kunming, 650033 China; 5 Yunnan Minzu University, Kunming, 650504 China

**Keywords:** Nanopore, Porin, RBD, SARS-CoV-2, Coronavirus, Vaccine

## Abstract

Coronavirus disease 2019 (COVID-19), caused by severe acute respiratory syndrome coronavirus 2 (SARS-CoV-2), seriously threatens human life and health. The correct folding and polymerization of the receptor-binding domain (RBD) protein of coronavirus in *Escherichia coli* may reduce the cost of SARS-CoV-2 vaccines. In this study, we constructed this nanopore by using the principle of ClyA porin polymerization triggered by the cell membrane. We used surfactants to "pick" the ClyA-RBD nanopore from the bacterial outer membrane. More importantly, the polymerized RBD displayed on the ClyA-RBD polymerized porin (RBD-PP) already displays some correct spatial conformational epitopes that can induce neutralizing antibodies. The nanostructures of RBD-PP can target lymph nodes and promote antigen uptake and processing by dendritic cells, thereby effectively eliciting the production of anti-SARS-CoV-2 neutralizing antibodies, systemic cellular immune responses, and memory T cells. We applied this PP-based vaccine platform to fabricate an RBD-based subunit vaccine against SARS-CoV-2, which will provide a foundation for the development of inexpensive coronavirus vaccines. The development of a novel vaccine delivery system is an important part of innovative drug research. This novel PP-based vaccine platform is likely to have additional applications, including other viral vaccines, bacterial vaccines, tumor vaccines, drug delivery, and disease diagnosis.

**Graphical Abstract:**

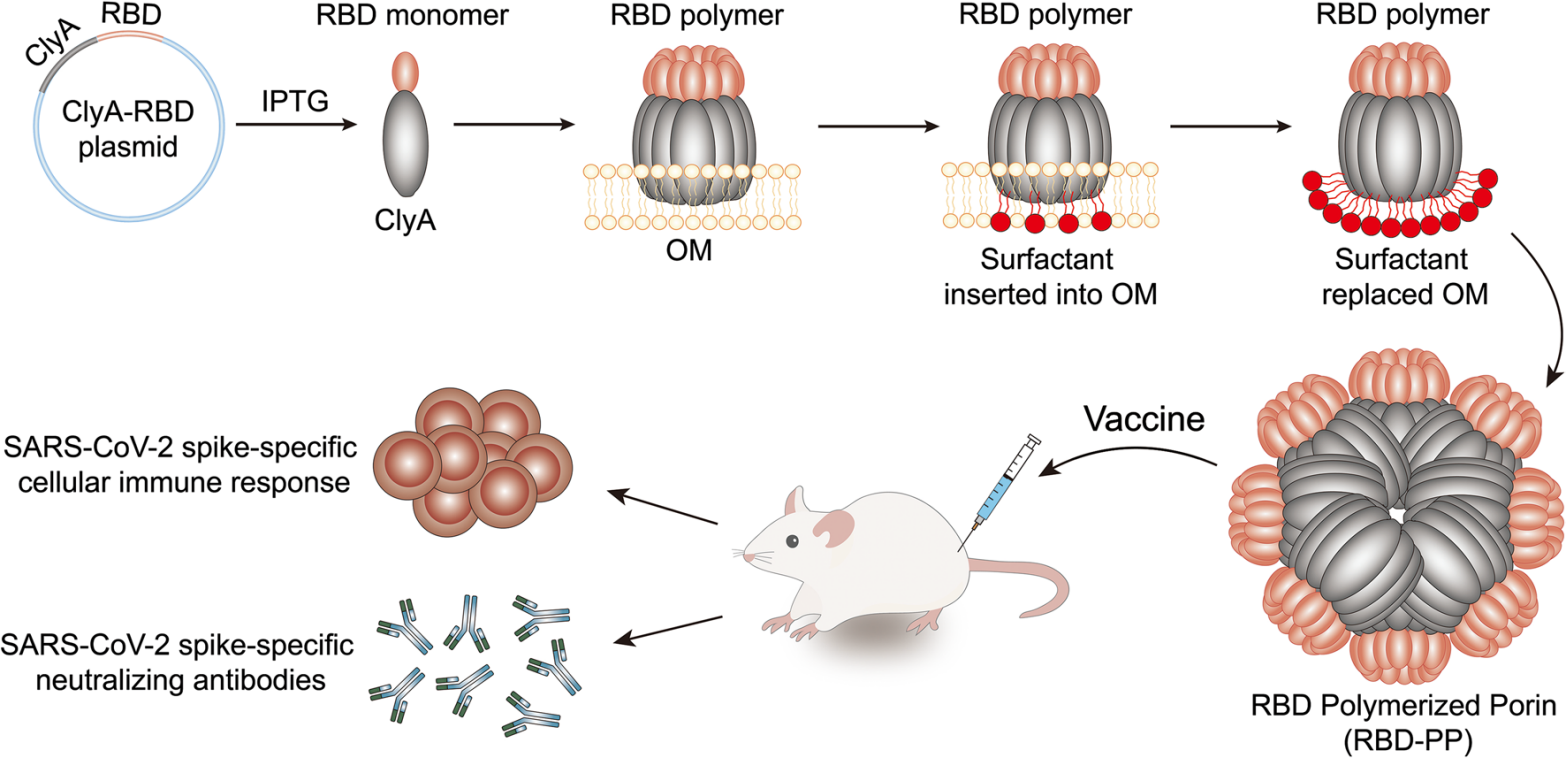

## Introduction

SARS-CoV-2 infection causes severe coronavirus disease 2019 (COVID-19) [1]. Inactivated vaccines [2], viral vector vaccines [3], subunit vaccines [4], mRNA vaccines [5], and DNA vaccines against SARS-CoV-2 have proven the effectiveness of different SARS-CoV-2 vaccines [6]. At present, some nanotechnologies that can efficiently display recombinant receptor-binding domain (RBD) protein have been gradually reported [7–13] and developed into the new nanovaccine against SARS-CoV-2, several nanovaccine formulations have also achieved antiviral effects. These nanovaccines can present antigens to the immune system at high densities, and antigen multimerization is important for the activation of low-affinity B cells [14, 15]. Relying on their nanoscale characteristics, nanovaccines induce superior lymph node targeting and uptake capacity by antigen-presenting cells [16]. They also exhibit excellent biocompatibility and safety.

*Escherichia coli* is a low-cost expression system. If an *E. coli* expression system could be used to manufacture a SARS-CoV-2 vaccine, the cost of vaccine production would be greatly reduced, which would facilitate global mass inoculation with SARS-CoV-2 vaccines. However, *E. coli* lacks a posttranslational modification system similar to those of higher organisms, so it cannot guarantee the folding of a protein to the correct conformation [17]. This has severely limited the application of the *E. coli* expression system to vaccine development. In addition, RBD exists in trimeric form in live SARS-CoV-2 [18], and it has been proven that RBD has better effects in multimeric form than in monomeric form [19, 20]. If the expression of the properly folded and polymerized RBD in *E. coli* for vaccine production can be optimized, the production cost of the SARS-CoV-2 vaccine will be greatly reduced.

ClyA is a porin on the outer membrane of *E. coli*. The monomer ClyA will polymerize on the outer membrane to form pores approximately 10–15 nm in size. In our previous work [21], we utilized ClyA to mimic the support structure of SARS-CoV-2 S2 protein for the RBD and achieved exterior exposure of the RBD on the self-assembled bacterial vesicles with high efficiency, leading to high exposure and polymerization of ClyA-RBD on bacterial membranes and the presentation of “ring-like” nanostructures on the vesicles (RBD-BBVs). These RBD-BBVs, when injected subcutaneously, can elicit SARS-CoV-2-specific humoral and cellular immune responses in mice. Despite the completion of this work on RBD-BBVs, however, BBVs derived from bacterial membranes present concerns about vaccine safety. To improve vaccine safety, it is necessary to break away from the bacterial membrane carrier to develop the key technology of the polymerization of ClyA alone. However, the technology of using ClyA alone to carry exogenous protein polymerization has not been developed, and ClyA must be supported by the bacterial membrane to form a polymer. If the self-assembled ClyA pore structure can directly carry exogenous proteins and form a nanoscale structure, this will be an ideal vaccine platform highly conducive to targeting lymph nodes, enabling antigen presentation and reducing systemic toxicity. The polymerization properties of ClyA are particularly suitable for presenting antigens that need to be polymerized, especially the RBD of SARS-CoV-2. Inspired by these properties, we wanted to construct a ClyA-RBD chimera and use the characteristics of ClyA self-polymerization to complete the polymerization of RBD, thereby constructing a safer polymerized coronavirus vaccine.

Although the polymeric nanopore structure of ClyA has been reported [22], there has not been any research on the use of ClyA nanopores as a vaccine delivery system based on antigen polymerization. Only the ClyA monomer with the correct conformation in the periplasm will be inserted into the outer membrane, thereby triggering the conformational change in the ClyA monomer [22, 23]. The ClyA monomer will move and adjust on the membrane after this conformational change, and multiple monomers will assemble into a polymer [22]. These conformational changes and assembly processes are not chaotic—they follow fixed rules for membrane insertion and assembly—and finally, ClyA will be assembled on the outer membrane into a structure with the C-terminus exposed outside the cell. Since the polymerization of the bacterial porin backbone of ClyA must be completed on the cell membrane, this pore structure is difficult to maintain after separation from the cell membrane [22, 24], which limits the development of ClyA nanopores as an independent delivery system. However, a large number of studies have proven that surfactants can also trigger the conformational rearrangement of ClyA monomers and the formation of nanopores [23, 25]. Inspired by this, we took advantage of this feature of ClyA to complete the self-assembly of the ClyA-RBD monomer from the membrane support to the surfactant support under the action of surfactants. In short, we used surfactants to "pick" the ClyA-RBD nanopore from the bacterial outer membrane. This was a great improvement compared to the RBD-BBV vaccine we previously reported [21].

Due to the importance of RBD polymerization in the development of coronavirus vaccines, in this study, we tried to develop and utilize ClyA nanopores as an RBD vaccine delivery system. We fused the ClyA protein, which is highly polymerized in *E. coli*, with RBD and achieved the correct folding and polymerization of RBD by exploiting the ability of ClyA to form nanopore structures on the outer membrane (OM) of *E. coli* [22, 26], finally forming ClyA-RBD polymerized porin (RBD-PP). A high degree of RBD polymerization was achieved in RBD-PP, and furthermore, the resulting nanostructures increased lymph node targeting and antigen uptake and processing by dendritic cells (DCs). In vivo immunization with RBD-PP induced the production of sufficient anti-SARS-CoV-2 neutralizing antibodies and T-cell responses and simultaneously activated memory T cells. This is the first report of a polymerized porin vaccine vector formed by the assembly of a bacterial protein and provides an approach to combating SARS-CoV-2 infection. Our findings provide not only a foundation for research on coronavirus vaccines but also a bacterial nanopore–based platform for vaccine research and development.

## Materials and methods

### Ethics and biosafety statement

All animal procedures were approved by the Institutional Animal Care and Use Committee of the Institute of Medical Biology, Chinese Academy of Medical Sciences (ethics approval number: DWSP202007002), and every effort was made to minimize animal suffering. All work with infectious SARS-CoV-2 was performed with approval under Biosafety Level 3 (BSL3) conditions by the Institutional Biosafety Committee of the Institute of Medical Biology. The BSL3 facilities have been designed to conform to the safety requirements recommended by the China National Accreditation Service for Conformity Assessment (CNAS) and the National Health Commission of the People’s Republic of China CNAS. Experiments with infectious virus were performed in a certified Class IIB biosafety cabinet in BSL3 [27].

### Mice, bacterial strains, cell lines, and virus

Specific pathogen-free BALB/c female mice (6–8 weeks old) were obtained from the Experimental Animal Center of the Institute of Medical Biology, Chinese Academy of Medical Sciences. All mice used in this study were in a healthy state and were raised in SPF animal facilities of the Institute of Medical Biology of the Chinese Academy of Medical Sciences, with free access to water and a standard chow diet. The *E. coli* strain BL21 was cultured in Luria–Bertani (LB) medium at 37 °C. The African green monkey kidney cell line Vero E6 (KCB 92017YJ) was obtained from the Conservation Genetics CAS Kunming Cell Bank (Kunming, China). These cells were maintained in Dulbecco’s modified Eagle’s medium (DMEM, Gibco) containing 10% fetal bovine serum (Gibco) at 37 °C in the presence of 5% CO_2_. The clinical isolates of SARS-CoV-2 wild-type and SARS-CoV-2 B.1.617.2 (Delta) variant were propagated in Vero E6 cells, and the successful propagation of SARS-CoV-2 was confirmed via qRT–PCR, sequencing, transmission electron microscopy (TEM), and titration via plaque assay (10^6^ plaque-forming units/mL) [28].

### Preparation of RBD-PP

The plasmid pThioHisA was purchased from Invitrogen, Inc. The RBD (Asn331-Val524, YP_009724390) was ligated to the C-terminus of the ClyA protein (AAL55667), with GS as the linker. The DNA fragment encoding the fusion protein ClyA-RBD was optimized and subcloned into pThioHisA using the restriction endonucleases *BamHI* and *SalI*, and the recombinant plasmid expressing the ClyA protein (AAL55667) was prepared in the same manner. Positive plasmids were confirmed by restriction digestion analysis and sequencing. The recombinant plasmids were transformed into *E. coli* BL21 and then inoculated into LB medium. When the optical density at 600 nm (OD_600_) of the bacterial culture reached 0.4–0.6, the bacterial culture was added to 1 mM isopropyl-β-d-1-thiogalactopyranoside (Solarbio) to induce the expression of the recombinant fusion protein at 30 °C overnight. A total of 2 mM EDTA·2Na (Sigma) was added to the sample the next day; after 2 h of incubation, the sample was centrifuged at 14,000×*g* at 4 °C for 30 min to collect bacterial cells. The cells were sonicated 5 times (power: 300 W, sonication time: 20 s, interval: 20 s) and then centrifuged at 6000×*g* and 4 °C for 10 min to remove unbroken bacteria. The supernatant was centrifuged at 100,000×*g*, and the pellet was resuspended in Hanks’s buffered saline solution (HBSS) (Servicebio). N-Lauryl sarcosyl (Sigma–Aldrich) was added to a final concentration of 2% and incubated at room temperature for 30 min, followed by centrifugation at 15,000×*g* for 30 min to induce precipitation as a component of the bacterial outer membrane. The precipitate was resuspended in phosphate-buffered saline (PBS) at 5 mg/ml and then incubated with 1% Triton X-100 or n-dodecyl-D-maltoside (DDM) at 15 °C for 2 h to extract ClyA-RBD nanopores from the bacterial outer membrane. The outer membrane was gradually replaced by surfactant insertion, and then ClyA-RBD nanopores were collected by ultracentrifugation at 130,000×*g* for 30 min. The precipitate was resuspended in PBS, stirred overnight at 37 °C, and purified by gradient centrifugation with OptiPrep™ (STEMCELL) medium. The concentrations of OptiPrep™ in HBSS for the gradient layers from bottom to top were 45%, 35%, 30%, 25%, 20%, 15%, and 10%. Samples were collected by centrifugation at 130,000×*g* for 4 h. The samples were stained with a Fast Silver Stain Kit (Beyotime). The particle sizes of the samples were analyzed using dynamic light scattering (DLS) (Zetasizer Nano ZS, Malvern Instruments). The protein concentration of boiled RBD-PP or PP was detected using a Bradford kit (Sangon Biotech) under denaturing conditions.

### Endotoxin removal

Endotoxin was removed using Pierce™ High Capacity Endotoxin Removal Spin Columns (Thermo). The sample (1 mL) was added to a buffer (pH 7.2) containing 10 mM sodium phosphate, 0.15 M sodium chloride, and 0.05% sodium azide and incubated at 22 °C with gentle end-over-end mixing for 1 h. The column was centrifuged at 500×*g* for 1 min to collect the sample. Endotoxin levels were measured using the Thermo Scientific Pierce LAL Chromogenic Endotoxin Quantitation Kit (Thermo).

### Western blot

Samples were run on a 12% sodium dodecyl sulfate (SDS)–polyacrylamide gel electrophoresis (PAGE) gel and transferred onto polyvinylidene fluoride membranes. The membranes were blocked in 5% milk for 45 min at room temperature. The anti-SARS-CoV-2-S1 rabbit monoclonal antibody (Sino Biological) and the goat anti-rabbit immunoglobulin G (IgG)-horseradish peroxidase (HRP) (Sino Biological) were used as the primary antibody (1:2000 dilution) and secondary antibody (1:10,000 dilution), respectively, for the identification of proteins expressed in the PP samples. The sera of immunized mice and goat anti-mouse IgG-HRP (Sino Biological) were used as the primary antibody (1:1000 dilution) and secondary antibody (1:10,000 dilution), respectively, for the identification of SARS-CoV-2 spike recombinant RBD protein (Sino Biological) and SARS-CoV-2 S1 protein (Sino Biological). Blots were visualized by using Clarity™ Western ECL Substrate (Bio–Rad).

### Polymerization analysis

The PP and RBD-PP samples were denatured through boiling at 95 °C for 5 min. Nondenatured samples did not undergo the above treatment. The samples were separated by 15% SDS–PAGE and stained with the Fast Silver Stain Kit (Beyotime).

### Transmission electron microscopy (TEM)

TEM was performed as previously described [29]. The samples were added to a carbon-coated copper grid and negatively stained for 30 s with 2% (wt/vol) uranyl acetate, and TEM images were collected using a transmission electron microscope (Hitachi).

### ClyA-RBD monomer preparation, binding analysis with hACE2 and neutralizing antibodies

As mentioned earlier [26], the osmotic shock method was used to obtain periplasmic components, and the ClyA-RBD monomer was purified. ClyA-RBD monomer and RBD-PP (3 μg/ml) were used to coat 96-well flat-bottomed plates (Corning), which were then incubated at 4 °C overnight. The plates were then washed with PBS containing 0.1% Tween 20 (PBST) three times, blocked with 1% bovine serum albumin (BSA) (Sigma) in PBST for 1 h at room temperature, added to serially diluted His-tagged hACE2 (Sino Biological) and incubated at 37 °C for 1 h, added to HRP-conjugated His antibody (Sino Biological, 0.2 µg/ml) at room temperature for 1 h, and finally added to 3,3′,5,5′-tetramethylbenzidine (TMB) (Solarbio) to observe the reaction. In the analysis of binding by neutralizing antibodies, the SARS-CoV-2 spike neutralizing antibodies MM43, MM57, R001, R004, D001, and D002 (Sino Biological, 0.1 µg/ml) were used as the primary antibodies, and HRP-conjugated antibodies against human IgG, mouse IgG, and rabbit IgG (Sino Biological, 1:10,000) were used as the secondary antibodies. After the reaction was terminated with H_2_SO_4_, a microplate reader (Thermo) was used to measure the OD450.

### Live imaging in vivo

RBD-PP and RBD were labeled with Cy7-NHS (Amersham Biosciences). Excess free dye was removed by a Sephadex G-25 PD 10 desalting column (GE Healthcare). The protein and Cy7 concentrations were determined by measuring the absorbance at 280 nm and 747 nm. Cy7-labeled RBD or RBD-PP (10 µg) or the same amount of free Cy7 was administered to mice via intramuscular injection. Twelve hours after the injection, the mouse's heart, liver, spleen, lung, kidney and lymph node were collected and imaged with an In Vivo FX PRO imaging system (Bruker) with an excitation filter at 760 nm and an emission filter at 790 nm. Molecular imaging software was used to determine the radiant efficiency and relative fluorescence intensity.

### BMDC uptake in vitro

Bone marrow–derived dendritic cells (BMDCs) isolated from murine femurs and tibias were cultured in plates as previously described [30]. Either RBD-PP or RBD was added to each well at 0.05 mg/ml, followed by incubation at 37 °C in 5% CO_2_ for 4 h. The incubated BMDCs were stained with the anti-mouse CD11c monoclonal antibody (BioLegend) at 4 °C for 30 min. After the excess antibody was removed by centrifugation, BMDCs were fixed with 4% paraformaldehyde, permeabilized with 0.2% Triton X-100, blocked with 2% BSA (Sigma), incubated with rabbit anti-SARS-CoV-2-S1 monoclonal antibody (Sino Biological, 1:200 dilution) at 37 °C for 2 h, incubated with goat anti-rabbit IgG-FITC (Proteintech, 1:50 dilution) at 37 °C for 1 h, and finally stained with 4′,6-diamidino-2-phenylindole (DAPI) (Meilunbio). The stained BMDCs were imaged using an ImageXpress Micro Confocal System (Molecular Devices).

### Antigen uptake and processing and presentation analysis in vivo

After 10 µg of Cy7-labeled RBD or RBD-PP was injected subcutaneously into mice for 12 h, the lymph nodes of the mice were collected and treated with 0.5 mg/mL collagenase I (Sigma) at 37 °C for 1 h. Lymphocytes in the lymph nodes were isolated by passing through a 70 μm cell strainer (FALCON) and centrifuged at 500×*g* for 5 min. The collected cells were stained with anti-mouse CD11c, CD86, CD80 and MHC-II (BioLegend) monoclonal antibodies, incubated at 4 °C for 30 min, and centrifuged to remove excess antibodies. The cells were analyzed by a CytoFLEX flow cytometer (Beckman Coulter), and data analysis was performed using FlowJo software.

### Germinal center responses

BALB/c mice were immunized subcutaneously with vaccine formulations containing 50 µg of RBD-PP and RBD mixed with alum, and PBS mixed with alum was used as the control. Ten days after injection, cell suspensions prepared from lymph nodes were stained with anti-mouse CD3, CD4, CXCR5, PD-1, CD19, B220, CD95 and GL7 (BioLegend) monoclonal antibodies and analyzed by a CytoFLEX flow cytometer (Beckman Coulter).

### Humoral immune response analysis

RBD-PP (50 µg, 5 µg, 0.5 µg), PP (50 µg, 5 µg, 0.5 µg) and ClyA-RBD monomer (RBD, 50 µg, 5 µg, 0.5 µg) were used to immunize 6- to 8-week-old female BALB/c mice on days 0, 14, and 28 by subcutaneous injection with alum (Thermo) as the adjuvant. Sera were collected from the mice on days 7, 21, and 35. Blocked enzyme-linked immunosorbent assay (ELISA) plates were precoated with SARS-CoV-2 RBD protein (Sino Biological) at 2 µg/ml overnight at 4 °C and used to analyze diluted serum samples that were sequentially diluted twofold with blocking buffer starting at 1:100 and incubated at 37 °C for 1 h. These samples were treated with biotin-conjugated anti-mouse IgG (Invitrogen, 1:5000 dilution), anti-mouse IgG1 (BioLegend, 1:8000 dilution), and anti-mouse IgG2a (BioLegend, 1:8000 dilution) and incubated at room temperature for 1 h. Next, streptavidin-HRP (BioLegend, 1:3000 dilution) was added and incubated at room temperature for 45 min. Finally, TMB (Solarbio) was added to observe the reaction. After the reaction was terminated with H_2_SO_4_, a microplate reader (Thermo) was used to measure the OD450. The endpoint titer was defined as the highest reciprocal dilution of serum to give an absorbance greater than 2.1-fold of the background values [20].

### Evaluation of antibody blocking S1/ACE2 binding

Diluted serum samples were added to ELISA plates in which each well contained 40 ng/ml SARS-CoV-2 RBD protein (Sino Biological) and incubated at 37 °C for 1 h. Next, His-tagged hACE2 (Sino Biological, 2 µg/ml) was added and incubated at 37 °C for 1 h; HRP-conjugated His antibody (Sino Biological, 0.2 µg/ml) was added and incubated at room temperature for 1 h; and TMB (Solarbio) was added to observe the reaction. After the reaction was terminated with H_2_SO_4_, a microplate reader was used to measure the OD450.

### Neutralization assays

The neutralization effect of antisera on SARS-CoV-2 infection was evaluated as previously described [31] in a biosafety level 3 facility. Vero E6 cells were seeded in 96-well plates and grown overnight. Mouse sera were serially diluted twofold with DMEM starting at 1:16. One hundred TCID_50_ (50% tissue culture infectious dose) of SARS-CoV-2 wild-type (WT) virus and SARS-CoV-2 B.1.617.2 (Delta) variant were preincubated with an equal volume of diluted serum and incubated at 37 °C for 1 h. The incubated mixture was then added to Vero E6 cells. The cytopathic effect (CPE) was recorded under a microscope on the 3rd day after infection. The neutralization titer NT_50_ was defined as the reciprocal of the serum dilution at which SARS-CoV-2 infectivity was reduced by 50%.

### Cellular immune response analysis

Fourteen days after the last immunization, the mice were sacrificed. The spleens were harvested, and splenocyte suspensions were isolated by passing through a 70 μm strainer. To measure the percentages of proliferating T cells in response to antigenic restimulation, splenocytes (1 × 10^6^/well) were cultured in 96-well plates and stimulated with SARS-CoV-2 RBD protein (2 μg/ml, Sino Biological). After incubation at 37 °C in 5% CO_2_ for 48 h, the splenocytes and cell culture media were collected by centrifugation at 500×*g*. The cells were stained with anti-mouse CD3, CD4 and CD8 monoclonal antibodies (BioLegend) and analyzed via flow cytometry. The cell culture media were used to determine cytokine levels with mouse GzmB, IFN-γ, and IL-2 ELISA kits (Invitrogen) according to the manufacturer’s instructions. Intracellular cytokine staining was performed to analyze SARS-CoV-2-specific CD4^+^ T helper cell and polyfunctional CD8^+^ T cell populations. Splenocytes (1 × 10^6^/well) were stimulated with SARS-CoV-2 RBD protein (2 μg/ml) for 1 h, and then 5 μg/ml brefeldin A (BioLegend) was added and incubated for 4–6 h to block the intracellular secretion of cytokines. These splenocytes were washed with PBS and stained with anti-mouse CD3, CD4 and CD8 monoclonal antibodies (BioLegend) at 4 °C for 30 min. After staining of the surface markers, splenocytes were treated with a fixation/permeabilization kit (BD Biosciences) according to the manufacturer’s protocol for anti-mouse IFN-γ, TNF-α, IL-2, and IL-4 monoclonal antibody (BioLegend) staining. The stained splenocytes were analyzed in a CytoFLEX flow cytometer (Beckman Coulter).

### Analysis of memory T cells

Fourteen days after the last immunization, the mice were sacrificed. The spleens were harvested, and splenocyte suspensions were isolated by passing through a 70 μm strainer. For the analysis of memory T cells, splenocytes were stained with anti-mouse CD3, CD4, CD8, CD44 and CD62L monoclonal antibodies (BioLegend) at 4 °C for 30 min. The stained splenocytes were analyzed in a CytoFLEX flow cytometer (Beckman Coulter).

### Enzyme-linked immunospot assay (ELISpot)

After splenocytes (3 × 10^5^/well) were stimulated with SARS-CoV-2 RBD protein (2 μg/ml) for 20 h, antigen-specific T-cell responses were evaluated using the mouse IFN-γ ELISpot^PLUS^ kit (Mabtech) and mouse IL-4 precoated ELISpot kit (Dakewe Biotech) according to the manufacturer’s instructions. The spots were counted using an ELISpot reader (AID).

### Safety evaluation

The body temperature and body weight of the mice were monitored and recorded once every 2 days during the immunization period. Seven days after the third immunization, the kidneys, hearts, livers, and spleens of the immunized mice were collected, and parts of the collected samples were taken for pathological analysis. These samples were fixed with 10% formalin for 24 h and sectioned for hematoxylin and eosin (H&E) staining. H&E images were acquired using a phase-contrast inverted fluorescence microscope (TS2, Nikon). To further evaluate the safety of RBD-PP, 50 μg/50 µl of RBD-PP or 50 µl of PBS was subcutaneously injected into BALB/c mice, and the IL-6 and IFN-γ levels in serum samples at 2 and 24 h after injection were detected using mouse IL-6 and IFN-γ ELISA kits (Invitrogen). The serum levels of alanine aminotransferase (ALT), aspartate transaminase (AST), alkaline phosphatase (ALP) and urea nitrogen (BUN) were detected using an automated analyzer (Chemray-800) at 6 h and 7 days after injection.

### Statistical analysis

Statistical analyses were performed with Prism 8.0 (GraphPad software). Specific methods used to assess the statistical significance of differences between groups are indicated in the legend. *P < 0.05, **P < 0.01, ***P < 0.001, ****P < 0.0001, ns, no significance.

## Results

### ClyA porin carrying RBD forms RBD-PP

**Fig. 1:**
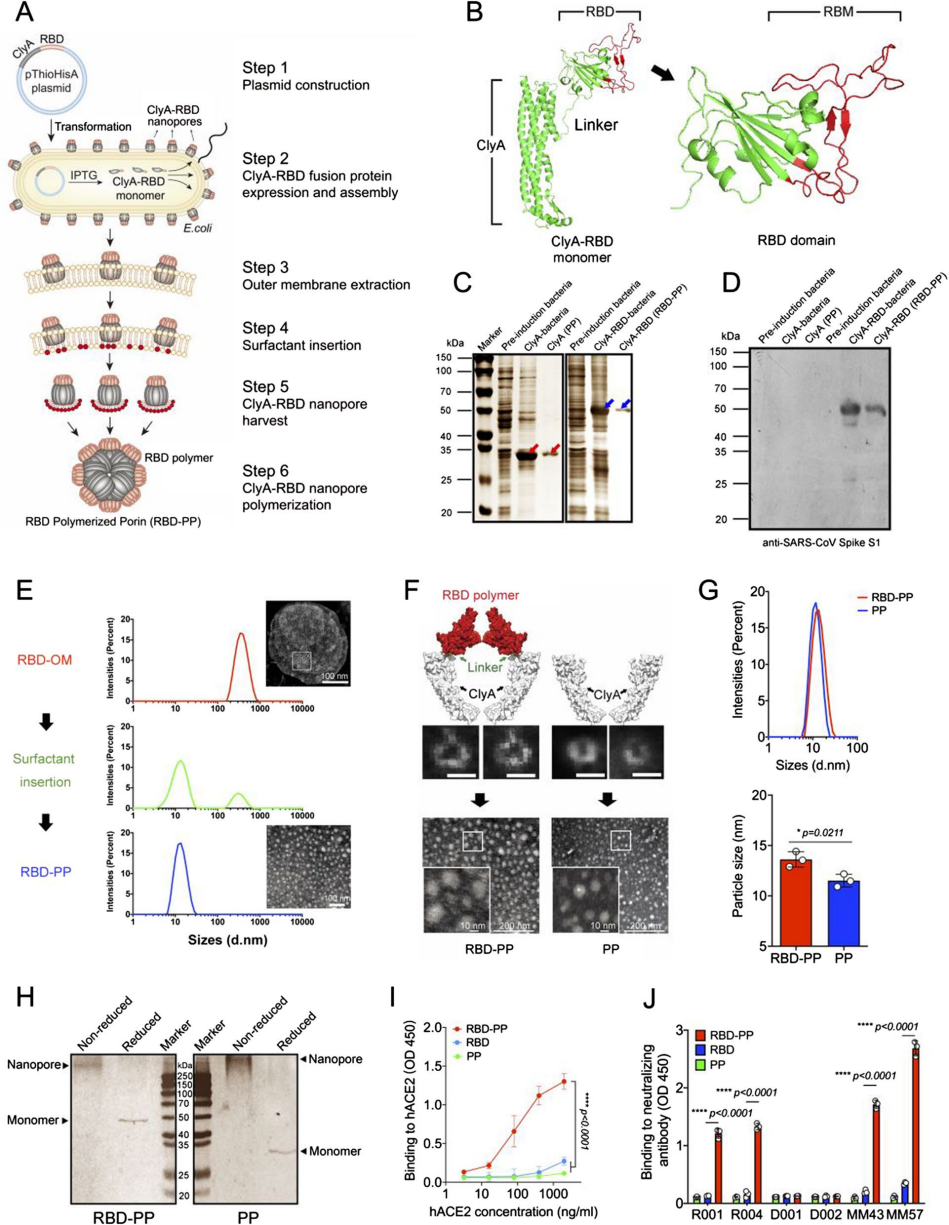
ClyA porin carrying RBD forms RBD-PP. **A** Schematic diagram of the preparation of RBD-PP. **B** Schematic diagram of the ClyA-RBD fusion protein. Red represents the receptor-binding motif (RBM). **C** After SDS–PAGE, the total bacterial protein products and purified PP and RBD-PP samples were analyzed by silver staining. The red arrow indicates ClyA, and the blue arrow indicates ClyA-RBD. **D** Western blotting was performed using an anti-SARS-CoV-2 spike S1 antibody. **E** The distributions of the particle sizes of the bacterial outer membrane overexpressing ClyA-RBD (RBD-OM), the intermediate process of the surfactant being gradually inserted into the outer membrane and self-assembly, and the purified RBD-PP. In a representative TEM image of RBD-OM, the small white circles in the white box mark the nanopores on the outer membrane, bar = 100 nm. **F** PyMOL was utilized to visualize homology modeling results; at the top is a schematic representation of the polymeric chains formed by monomers. Red represents RBD, and green represents the linkers. In the middle is the sporadic nanopore structure observed under TEM, bar = 10 nm. At the bottom is the final stable RBD-PP and PP electron microscope image, and the bottom left corner is an enlarged image of the area in the white frame. **G** The top panel shows the DLS analysis of RBD-PP and PP, and the bottom panel shows the statistical analysis of particle size (n = 3). **H** Denatured and nondenatured samples of ClyA and ClyA-RBD were analyzed by SDS–PAGE. Arrows indicate the positions of the denatured monomer and nondenatured polymer. **I** The binding ability of RBD-PP with hACE2 (n = 3). **J** Six monoclonal antibodies that can neutralize SARS-CoV-2 were allowed to react with RBD-PP (n = 3). The half-maximal inhibitory concentrations (IC_50_ values) of the neutralizing antibodies MM43, MM57, R004, R001, D001, and D002 were 1.41 μg/ml, 0.41 μg/ml, 0.234 μg/ml, 0.11 μg/ml, 0.646 μg/ml, and 46.76 μg/ml, respectively. Data are shown as the mean ± SD. Statistical significance was calculated via one-way ANOVA (**J**), two-way ANOVA (**I**) or Student’s *t* test (**G**) to obtain *p* values: ****p < 0.0001, and *p < 0.05

We expressed the fusion protein of a bacterial porin, ClyA, with SARS-CoV-2 RBD in *E. coli* and achieved highly ordered assemblies of the fusion protein ClyA-RBD by utilizing the self-assembly function of ClyA on the outer membrane. We extracted the bacterial outer membrane and gradually inserted surfactants to replace the bacterial outer membrane while maintaining the nanopore structure of ClyA. We obtained RBD-modified nanopores, called ClyA-RBD nanopores, which finally polymerized into stable RBD-PP (Fig. 1A). The endotoxin level of purified RBD-PP was < 0.25 EU/ml. The correct folding of the protein is very important for vaccine design. The modeling results show that ClyA and RBD fold separately without interfering with each other and form their respective correct protein conformations, and the RBD and receptor-binding motif (RBM) in it are completely exposed [21] (Fig. 1B). Compared with bacterial whole cells, the ClyA polymerized porin (PP) and ClyA-RBD polymerized porin (RBD-PP) we harvested after purification exhibited single protein bands (Fig. 1C). The S1 antibody against SARS-CoV-2 was used to verify that the obtained RBD-PP contained RBD protein (Fig. 1D). The nanopores formed on the bacterial outer membranes that overexpressed ClyA-RBD, and analysis of the particle size of the ClyA-RBD outer membrane (RBD-OM) by DLS revealed a peak at ~ 384.9 nm. The existence of these nanopores on the bacterial membrane can be observed by TEM. After surfactant insertion, the outer membrane is gradually replaced, and the self-assembly of ClyA is realized [23, 25]; hence, we observed two DLS peaks, one at approximately 13.14 nm and one at approximately 324.9 nm, followed by the eventual formation of stable RBD-PP, which appeared at approximately 13.9 nm in the DLS data (Fig. 1E). When ClyA completes self-assembly, it carries RBD to form a polymerized pore structure (Fig. 1F red part). We observed very few possible single-pore structures under TEM, and the morphology of RBD-PP was different from that of PP. It can be seen that RBDs may polymerize together. The single nanopore structure intermediate ultimately forms a stable polymerized porin. The TEM images show that RBD-PP is larger than PP with rougher edges, which may be due to the RBD polymerization display (Fig. 1F). DLS analysis also proved that the particle size of RBD-PP is larger than that of PP (Fig. 1G). To prove the polymeric state of RBD-PP, we performed SDS–PAGE on denatured or nondenatured samples. The results confirmed the polymeric structures of both RBD-PP and PP (Fig. 1H). Most importantly, polymerized RBD-PP binds hACE2 more strongly than the RBD monomer does (Fig. 1I). These results show that RBD-PP exposes a high degree of RBD polymer on the surface and that the RBD polymer binds hACE2 more strongly than the RBD monomer does. We speculate that the enhanced binding ability of RBD-PP with hACE2 is due to the formation of a spatial conformation with stronger affinity for ACE2 during the polymerization of the RBD monomer accompanied by ClyA. Because the RBD expressed in *E. coli* lacks glycosylation modification, the spatial conformation of the RBD expressed in *E. coli* is particularly important for the ability to induce neutralizing antibodies against the virus. To analyze the conformation of the polymerized RBD displayed on RBD-PP, we tested the recognition of RBD-PP by 6 kinds of neutralizing antibodies that have been shown to recognize the spatial epitope of the virus. The experimental results proved that 4/6 antibodies can recognize RBD-PP, whereas these antibodies have almost no ability to recognize RBD monomer (Fig. 1J). This result indicates that the polymerization display of RBD on RBD-PP may form a spatial conformation capable of inducing neutralizing antibodies that is not formed by RBD monomers lacking glycosylation. Therefore, the results suggested that the spatial conformation formed by the polymerized RBD fold is more correct than that of the monomer.

### RBD-PP targets lymph nodes and enhances antigen processing

**Fig. 2:**
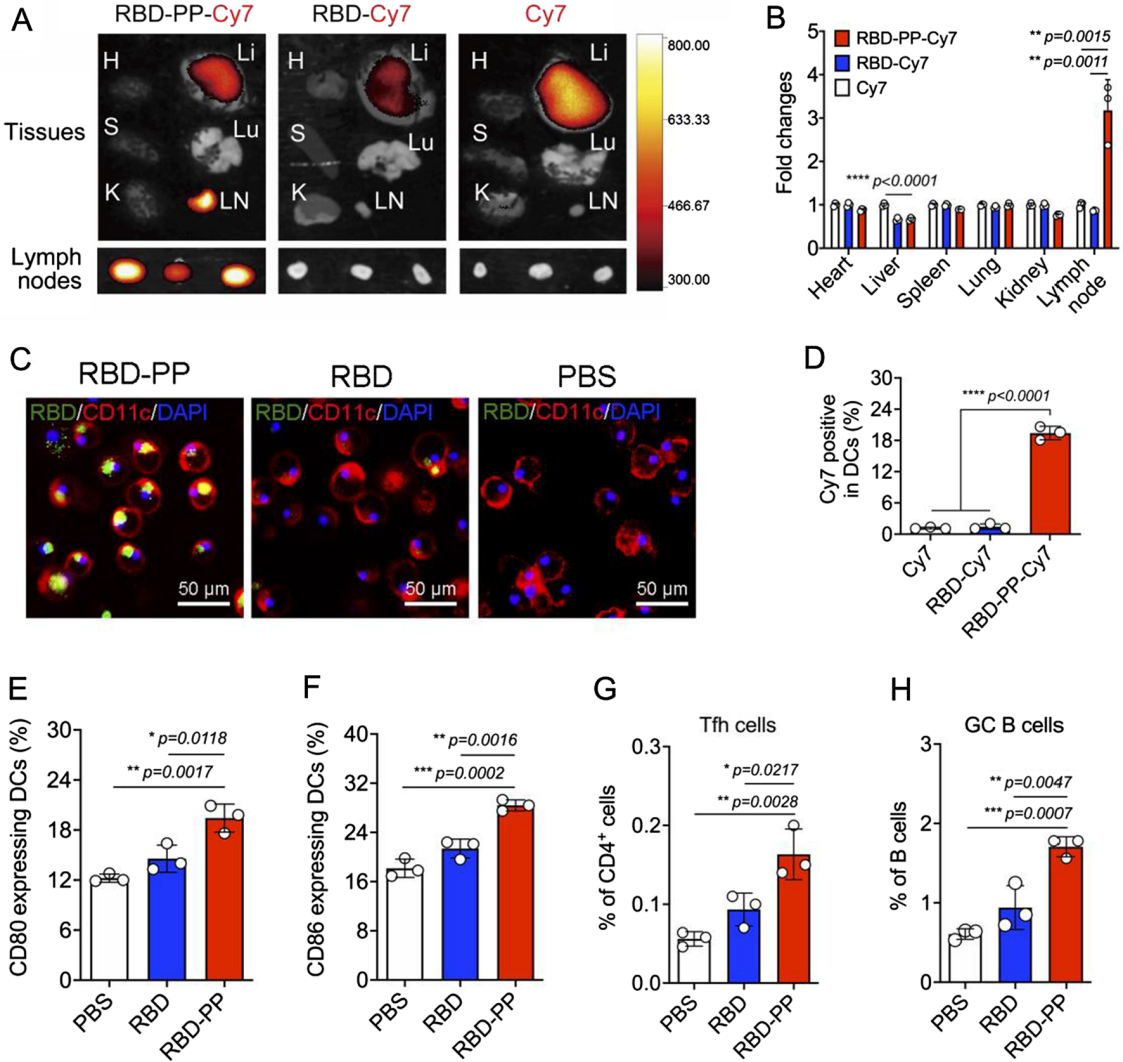
RBD-PP targets lymph nodes and enhances antigen processing. **A** Twelve hours after the subcutaneous injection of 10 µg Cy7-labeled samples for each group, the heart (H), liver (Li), spleen (S), lung (Lu), kidney (K) and inguinal lymph nodes (LN) were collected and observed by live imaging. **B** The fold change of the fluorescence intensity relative to that in the free Cy7 control group (n = 3). **C** BMDC uptake. Representative images with green representing the antigen RBD, red representing CD11c, and blue representing DAPI-stained nuclei. **D** Twelve hours after the subcutaneous injection of 10 µg Cy7-labeled samples for each group, flow cytometry analysis of representative graphs of Cy7^+^CD11c^+^MHC-II^+^, (**E**) CD80^+^CD11c^+^MHC-II^+^, and (**F**) CD86^+^ CD11c^+^MHC-II^+^ cells in lymph nodes (n = 3). **G** Ten days after mice were immunized with 50 µg of different vaccines, the percentages of T follicular helper (Tfh) cells (CD3^+^CD4^+^CXCR5^+^PD-1^+^) and (**H**) germinal center (GC) B cells (CD19^+^B220^+^CD95^+^GL7^+^) in lymph nodes were measured by flow cytometry (n = 3). Data are shown as the mean ± SD. Statistical significance was calculated via one-way ANOVA (B, D-H) to obtain *p* values: ****p < 0.0001, ***p < 0.001, **p < 0.01, and *p < 0.05

Antigen delivery to the lymph nodes mediated by the vaccine delivery system can enhance the immune response elicited by the antigen, and in principle, a nanostructure can enter the lymph node efficiently. As expected, we found that RBD-PP can easily enter the lymph nodes due to its size of approximately 13.9 nm. The use of a fluorescence tracer in vivo showed that RBD-PP can enter lymph nodes more efficiently than RBD protein at 12 h after subcutaneous injection (Fig. 2A). Compared with free dyes, RBD-PP reduced liver fluorescence and effectively targeted lymph nodes while reducing systemic toxicity (Fig. 2B). In addition, we observed that compared to the free RBD form, the RBD-PP vaccine form greatly improved the uptake of RBD by BMDCs (Fig. 2C). To collect lymph nodes, we used flow cytometry to analyze the uptake and processing of antigens by DCs, and the results showed that Cy7-labeled RBD-PP was efficiently taken up by DCs (CD11c^+^MHC-II^+^) (Fig. 2D). At the same time, RBD-PP efficiently promoted a significant increase in the proportion of CD80^+^ (Fig. 2E) and CD86^+^ DCs (Fig. 2F). It has been proven that the RBD-PP vaccine effectively promotes both lymph node targeting and the uptake and processing of antigens by DCs, which is critical for improving vaccine efficacy. More importantly, RBD-PP significantly increased the proportion of T follicular helper (Tfh) cells (Fig. 2G) and germinal center (GC) B cells (Fig. 2H) in the lymph nodes of mice after a single immunization. Tfh cells can promote the production and maturation of neutralizing antibodies by helping B cells. These results show the advantage of RBD-PP in inducing an antibody response.

### RBD-PP can elicit anti-SARS-CoV-2 neutralizing antibodies

**Fig. 3:**
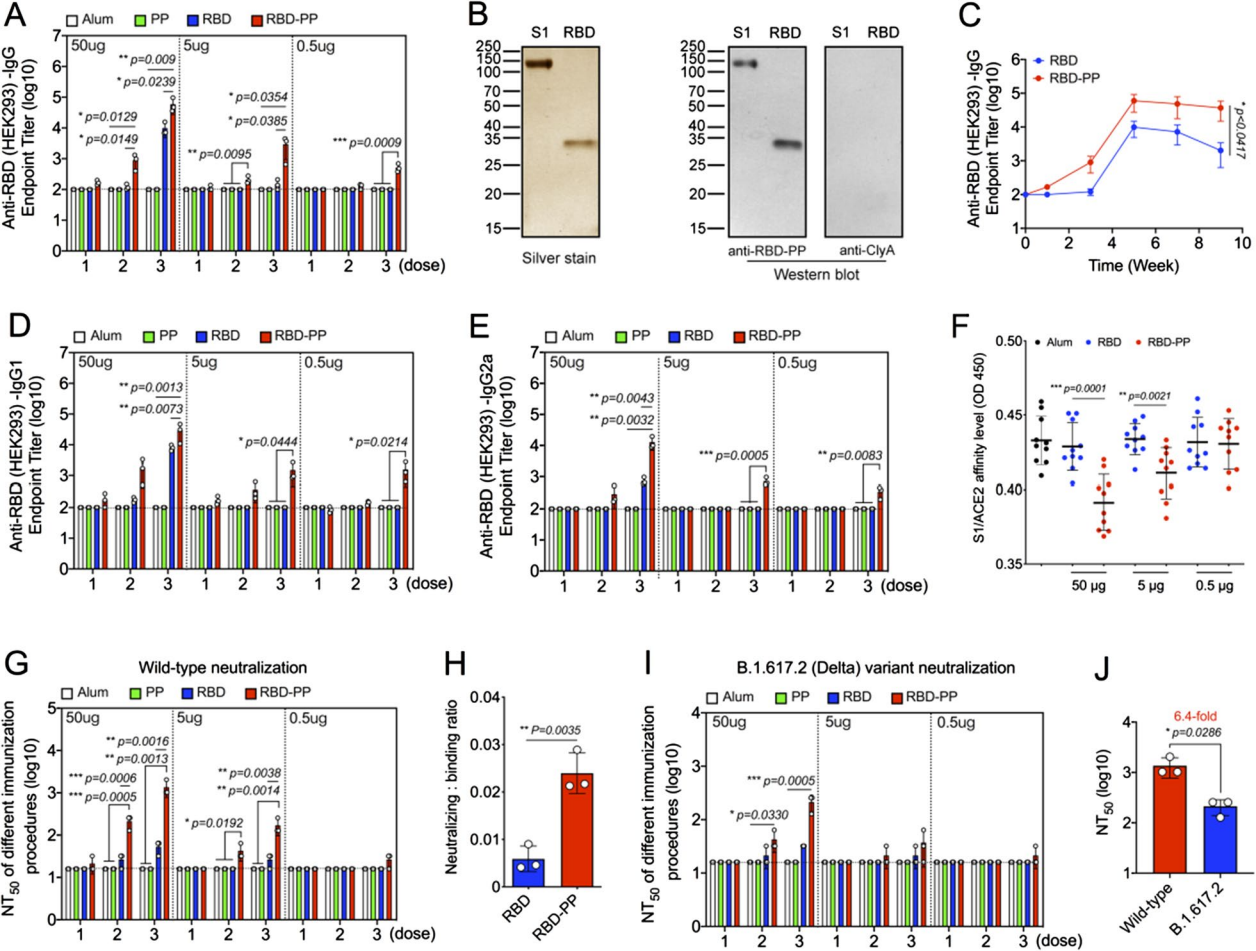
RBD-PP can elicit anti-SARS-CoV-2 neutralizing antibodies. Mice were vaccinated with the different vaccines on days 0, 14, and 28 of the experiment. Serum was collected 7 days after each vaccination. **A** Detection of anti-RBD IgG titers in mouse serum using ELISA (n = 3). **B** The left panel shows S1 and RBD proteins expressed in HEK293 cells. The right panel shows the results of a Western blot using diluted anti-RBD-PP and anti-ClyA serum (1:1000 dilution). **C** The time-course curve of the anti-RBD IgG titer elicited with different vaccines (n = 3). **D** Detection of anti-RBD IgG1 and (**E**) IgG2a titers in mouse serum using ELISA (n = 3). **F** Antiserum collected after three immunizations with different vaccines to block the binding of S1 and ACE2, denoted by the OD450 (n = 10). **G** The neutralizing activity of the immune serum was evaluated against SARS-CoV-2 wild-type (WT) virus, and the 50% neutralization titer (NT_50_) was determined (n = 3). **H** The ratio of the NT_50_ titer to the endpoint titer after three immunizations with 50 µg doses of vaccines (n = 3). **I** The neutralizing activity of the immune serum was evaluated against the SARS-CoV-2 B.1.617.2 (Delta) variant, and the NT_50_ was determined (n = 3). **J** Serum after three immunizations with 50 µg of RBD-PP and comparison of the NT_50_ between SARS-CoV-2 wild-type and SARS-CoV-2 B.1.617.2 (Delta) variant (n = 3). The dotted horizontal lines represent the lower limit of detection of the assay. Data are shown as the mean ± SD. Statistical significance was calculated via one-way ANOVA (**A**, **D**, **E**, **G**, **I**), two-way ANOVA (**C**) or Student’s *t* test (**F**, **H**, **J**) to obtain *p* values: ***p < 0.001, **p < 0.01 and *p < 0.05

Mice were vaccinated with RBD-PP on days 0, 14, and 28 of the experiment. Serum was collected 7 days after each vaccination. Anti-RBD IgG antibodies showed significant dose-dependent expression in the serum of mice vaccinated with RBD-PP. The 50 µg dose of RBD-PP induced a binding antibody titer of 10^3^ after the second immunization and as high as 10^4.8^ after the third immunization. RBD-PP at doses of 5 µg and 0.5 µg also induced significant binding antibody titers after the third immunization, reaching 10^3.5^ and 10^2.7^, respectively. The RBD monomer induced a significant binding antibody titer of only 10^4^ after the third immunization with a dose of 50 µg. This shows that compared to the RBD monomer, RBD-PP can more efficiently induce B-cell responses (Fig. 3A). To show that RBD-PP induced anti-RBD antibodies, the RBD-PP antiserum was used to identify S1 and RBD proteins expressed in HEK293 cells (Fig. 3B). The experimental results showed that the antibody induced by RBD-PP can correctly recognize S1 and RBD. In addition, at the 9th week after immunization with RBD-PP a high binding antibody titer was still maintained, but the binding antibody titer elicited by RBD monomer had dropped significantly (Fig. 3C). The IgG antibody subtypes IgG1 (Fig. 3D) and IgG2a (Fig. 3E) induced by RBD-PP were also stronger than those induced by RBD monomers. We next detected the ability of the RBD-PP antiserum to block S1/ACE2 binding. The experimental results showed that, compared with the effects of the RBD monomer, the serum of mice vaccinated with 50 μg or 5 μg RBD-PP blocked S1/ACE2 binding more effectively (Fig. 3F). Most importantly, the anti-RBD monomer antiserum collected after three immunizations with the highest dose (50 µg) did not induce a significantly different titer of neutralizing antibodies but showed only an increasing trend of neutralizing antibodies. In contrast, RBD-PP clearly induced neutralizing antibodies after immunization with a dose of 50 µg twice or with doses of 5 µg three times (Fig. 3G).

We also compared the ratio between the neutralizing antibody titer and the binding antibody titer of the serum after three immunizations with a dose of 50 μg [19]. The results showed that this titer ratio induced by RBD-PP increased significantly (Fig. 3H). This indicates that the increase in the neutralization titer of the RBD monomer is due to the increase in the titer of the binding antibody. Moreover, in the case of the same binding antibody, RBD-PP can induce stronger neutralizing antibodies, which shows that RBD-PP has more correct epitopes that can induce neutralizing antibodies. Therefore, RBD-PP induces more neutralizing antibodies, while the RBD monomer can induce only some binding antibodies with no neutralizing effect. Because viral mutation is a challenge to vaccine design, we also tested the neutralizing antibody titers of the RBD-PP antiserum against virus variants. The results showed that the antibodies induced by RBD-PP could also neutralize the SARS-CoV-2 B.1.617.2 (Delta) variant (Fig. 3I), but their ability to neutralize the virus decreased by 6.4-fold (Fig. 3J). This may be related to the mutation of the RBD site [32].

### RBD-PP can elicit a cellular immune response and memory T cells

**Fig. 4:**
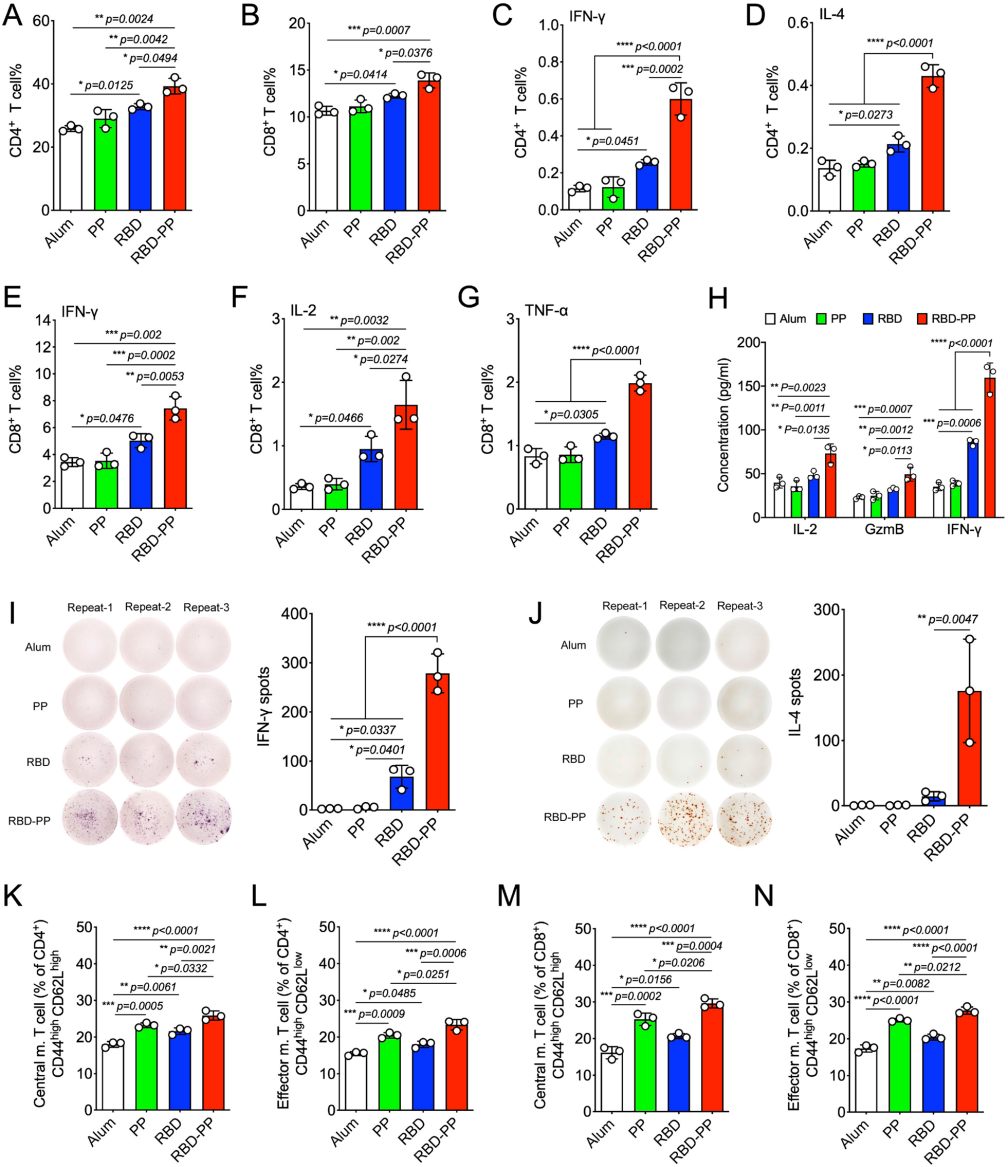
RBD-PP can elicit a cellular immune response and memory T cells. Mice were immunized three times with 50 µg doses of different vaccines. Fourteen days after the last immunization, splenocytes were isolated and stimulated with the RBD protein expressed in HEK293 cells. Flow cytometry was used to analyze the ratio of RBD-specific (**A**) CD4^+^ T (CD3^+^CD4^+^), (**B**) CD8^+^ T (CD3^+^CD8^+^), (**C**) Th1 (CD3^+^CD4^+^IFN-γ^+^), and (**D**) Th2 (CD3^+^CD4^+^IL-4^+^) cells. The proportion of multifunctional CD8^+^ T cells, which are defined as CD8^+^ T cells that release (**E**) IFN-γ, (**F**) IL-2, and (**G**) TNF-α, was detected (n = 3). **H** Splenocytes of immunized mice were stimulated with RBD protein expressed in HEK293 cells, and the culture supernatant after stimulation was used to analyze the release of cytokines (n = 3). **I** Representative ELISpot images of spots and bar graphs corresponding to IFN-γ- and (**J**) IL-4-secreting lymphocytes in RBD-stimulated splenocytes of immunized mice (n = 3). **K** Flow cytometry analyses of memory T cells in splenocytes from immunized mice. Bar graphs summarizing the flow cytometry results for CD4^+^ central memory T cells (CD3^+^CD4^+^CD44^high^CD62L^high^) (n = 3), (**L**) CD4^+^ effector memory T cells (CD3^+^CD4^+^CD44^high^CD62L^low^) (n = 3), (**M**) CD8^+^ central memory T cells (CD3^+^CD8^+^CD44^high^CD62L^high^) (n = 3), and (**N**) CD8^+^ effector memory T cells (CD3^+^CD8^+^CD44^high^CD62L^low^) (n = 3) in the spleen. Data are shown as the mean ± SD. Statistical significance was calculated via one-way ANOVA (**A**–**N**) to obtain *p* values: ****p < 0.0001, ***p < 0.001, **p < 0.01 and *p < 0.05

The cellular immune response plays an important role in the fight against SARS-CoV-2 infection [33]. Therefore, we examined the ability of RBD-PP to elicit cellular immune responses. Flow cytometry experiments on splenocytes from mice immunized with RBD-PP showed that all splenocytes had CD4^+^ (Fig. 4A) or CD8^+^ (Fig. 4B) cellular responses to the SARS-CoV-2 RBD protein. Both the CD4^+^ and CD8^+^ T-cell responses to RBD-PP are stronger than those to RBD monomer. In addition, RBD-PP induced the responses of Th1 (Fig. 4C) and Th2 (Fig. 4D) cells in T helper cell populations of spleen cells, and the mixed Th1 and Th2 immune response may produce comprehensive immune protection when the virus invades. SARS-CoV-2-specific cytotoxic T lymphocytes (CTLs) play important roles in virus clearance. Therefore, we also analyzed the activation level of CD8^+^ T cells. Compared with the RBD monomer, RBD-PP efficiently stimulated the activation of antigen-specific multifunctional CD8^+^ T cells, which was manifested by a significant increase in the proportion of CD8^+^ T cells released by IFN-γ (Fig. 4E), IL-2 (Fig. 4F), and TNF-α (Fig. 4G).

The splenocyte lymphocytes of mice immunized with RBD-PP secreted IL-2, GzmB, and IFN-γ effector molecules after stimulation by SARS-CoV-2 RBD proteins (Fig. 4H). We isolated lymphocytes from the spleen for ELISpot analysis. The results showed that RBD-PP induced a significant increase in IFN-γ-secreting (Fig. 4I) and IL-4-secreting (Fig. 4J) T cells in the spleen. These results show that RBD-PP can elicit systemic SARS-CoV-2-specific cellular immune responses. The induction of T-cell immune memory is the key to antiviral protection. Therefore, we also determined the ability of the RBD-PP vaccine to induce CD4^+^ T and CD8^+^ memory T cells in the spleen using flow cytometry. The results showed that compared with the RBD monomer, the RBD-PP vaccine induced higher CD4^+^ (Fig. 4K) and CD8^+^ (Fig. 4M) central memory T (CD44^high^CD62L^high^, Tcm) cells, which was consistent with the induction of effector memory T (CD44^high^CD62L^low^, Tem) cells among CD4^+^ (Fig. 4L) and CD8^+^ (Fig. 4N) cells. The induction of two memory T cells by the RBD-PP vaccine indicates that when the virus invades the body, an immediate antiviral effect will be provoked by Tem, and the activation of protective immunity will be stimulated through the proliferation and differentiation of Tcm. In summary, RBD-PP was able to induce a stronger T-cell immune response than the RBD monomer.

### RBD-PP has excellent in vivo safety

**Fig. 5:**
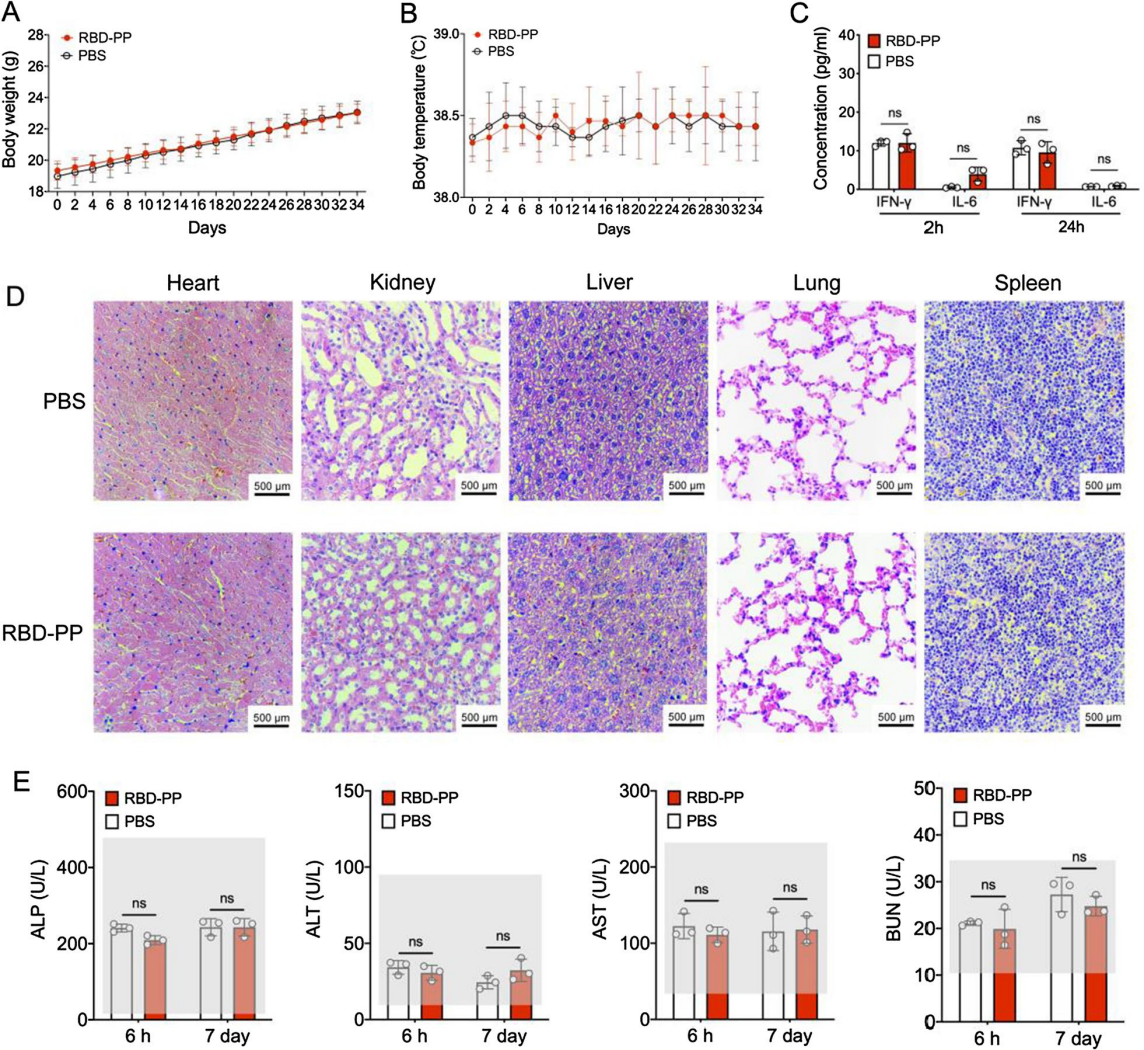
RBD-PP has excellent in vivo safety. **A** Body weight and (**B**) body temperature throughout the immunization program using 50 μg of RBD-PP (n = 3). **C** ELISA of the levels of the serum inflammatory cytokines IFN-γ and IL-6 2 h and 24 h after subcutaneous injection of 50 µg of RBD-PP (n = 3). **D** Representative images of HE-stained mouse tissue sections after the immunization program was completed. **E** Analysis of metabolic biochemical markers in serum. 6 h and 7 days after the mice were subcutaneously injected with RBD-PP (50 μg), the serum levels of ALP, ALT, AST, and BUN were detected (n = 3). The gray area represents the fluctuation range of the normal level. Data are shown as the mean ± SD. Statistical significance was calculated via Student’s *t* test (**C**, **E**): *ns* no significance

Finally, we preliminarily evaluated the animal safety of RBD-PP. We observed no significant body weight loss (Fig. 5A) or temperature fluctuation (Fig. 5B) in mice vaccinated with 50 μg RBD-PP during the immunization period. At 2 h and 24 h after subcutaneous injection of 50 μg RBD-PP, no significant increase in the serum inflammatory cytokines IFN-γ or IL-6 was observed (Fig. 5C). There were no significant tissue lesions (Fig. 5D) after the immunization program was completed. In addition, we tested the metabolic biochemical markers in serum 6 h and 7 days after RBD-PP immunization, and the experimental results showed that RBD-PP immunization did not cause significant liver or kidney damage (Fig. 5E). Therefore, we believe that RBD-PP has excellent in vivo safety at a 50 µg dose.

## Discussion

The keys to successful SARS-CoV-2 vaccination are efficient antigen processing presentation by APC cells, the induction of SARS-CoV-2 neutralizing antibodies, and the activation of T-cell responses and lasting immune memory. Although many SARS-CoV-2 vaccines satisfy these prerequisites and have entered clinical trials [6, 34], their production costs and outputs may limit the widespread application of these vaccines in the global pandemic. *E. coli* is the most productive and least costly protein expression system. Vaccines against SARS [35], dengue fever [29], Middle East respiratory syndrome [36], hepatitis E [37], influenza [38], and human papillomavirus [39] have been successfully expressed as nanostructures in *E. coli*. Therefore, the correct folding or assembly of viral antigens into nanostructures in *E. coli* could elicit potential immune protection, thus enabling the successful development of an inexpensive vaccine in *E. coli*.

The RBD of SARS-CoV-2 is the main mediator of the binding between viruses and the host cell receptor ACE2 [40]. It is also the main target of the SARS-CoV-2 vaccine [13, 41–43]. In its natural conformation, the RBD of SARS-CoV-2 shows a trimeric structure [18, 44]. The host immune response caused by this trimeric structure may not be identical to the host immune response caused by the monomeric structure. RBD dimers [20] and multimers [12, 19] have better immunoprotective ability than RBD monomer. Therefore, multimerization of RBD may be a key step in the design of SARS-CoV-2 vaccines. This study also found that RBD-PP has a stronger ability than monomers to bind hACE2, suggesting the importance of RBD polymers (Fig. 1I).

Because of their scale characteristics, nanovaccines can increase the effectiveness of lymph node targeting and antigen presentation [45]. Their unique structural features can enhance immunogenicity by presenting polyvalent antigens, stabilize antigens, and induce adjuvant activities [46]. A variety of organic and inorganic nanoparticles have been used in nanovaccines. For example, antigen-loaded nanomaterials, such as gold [47, 48], silica [49, 50], magnetosomes [51], poly(lactic‐co‐glycolic acid) [52], and polyethyleneimine [53, 54], have been successfully used in vaccines against viruses, tumors, bacteria, and inflammatory and immunoregulatory disorders [55]. However, the application of nanovaccines is often limited by their toxicity, bioincompatibility, instability, scale-up shortcomings, and low interbatch reproducibility. Biopolymerizable nanoparticles that rely entirely on biosynthesis are gradually emerging. The available techniques mainly utilize trimer tags, the C-terminal domain of T4 fibritin, three-helix bundles [56], self-assembling proteins (ferritin [57], lumazine synthase [58, 59], and encapsulin [60]), and self-assembling peptides [61–63] to carry large proteins or peptides to form regular polymers. Vaccines based on these polymeric structures have induced excellent immunogenic effects in terms of multiple aspects [58, 64]. Therefore, it is highly important to continue to develop nanovaccine delivery systems with antigen polymerization.

In the existing biopolymeric nanoparticles, researchers use self-assembled proteins such as I53-50 [19], ferritin [12, 65], mi3 [65], E2p [66], I3-01v9 [66], and modified tobacco mosaic virus [67] are used as nanoparticle carriers and gene fusion [19, 66], chemical coupling [67] and the SpyTag-SpyCatcher system [12, 65, 66] are used to display the RBDs expressed by the eukaryotic system on the surface of nanoparticles. These RBD-displaying nanoparticles significantly improve the immune effect compared with that elicited by RBD monomers. Unlike these nanoparticles, ClyA-RBD polymerized porin nanostructures are formed by the self-assembly of prokaryotically expressed fusion proteins in bacterial cells. These RBD-displaying nanoparticles require a complex production process, including two expression systems, separate purification steps, and in vitro assembly steps before final purification. In contrast, we used a single prokaryotic expression system to simplify the production process and reduce the production cost. Moreover, animal immune models also showed that ClyA-RBD polymerized porin nanostructures elicited significant immune effects. The polymerized porin nanostructures can thus avoid the dependence on mammalian expression systems in RBD display and promote the development of inexpensive vaccines.

The site of ACE2 binding to the RBD does not have a glycosylation modification, so it is theoretically possible to design RBD-based vaccines in prokaryotic systems [31]. Here, we used the polymerization function of ClyA to achieve the regular polymerization of RBD in *E. coli*. The RBD displayed by polymerization on RBD-PP showed more spatial conformational epitopes similar to those of the natural RBD than the RBD monomer did, which would be more conducive to induce neutralizing antibody production, thus avoiding the incorrect folding of E. coli–expressed RBD. The ClyA protein naturally polymerizes in *E. coli* outer membrane vesicles (OMVs) [22, 26] and is widely used in genetic engineering for the translocation of heterologous proteins to modify OMVs [68, 69]. OMVs contain many toxic proteins, so there is a safety issue with using OMVs as a vaccine vector. If this type of self-assembled ClyA nanopore can directly carry heterologous proteins and form nanometer-scale structures, the self-assembled ClyA nanopore (approximately 14 nm) would be an ideal vaccine platform because it can easily target lymph nodes and reduce toxicity to other organs (Fig. 2A) [45, 70], stimulating stronger antigen presentation. More importantly, the polymerization of ClyA is particularly suitable for the presentation of antigens that need to be polymerized, especially the RBD of SARS-CoV-2. No technology utilizing ClyA nanopores as a vaccine vector has been developed before.

We developed ClyA-RBD by the fusion of ClyA, a porin that is polymerized in bacteria, with full-length RBD. The fusion protein ClyA-RBD is abundantly expressed in the cytoplasm of *E. coli* and can migrate to the periplasm and the outer membrane [26], where the oxidative environment favors its proper folding [17]. ClyA undergoes a conformational rearrangement triggered by the lipid membrane and assembles into pores on the bacterial outer membrane [24, 71]. RBD forms a polymeric structure with ClyA, and ClyA-RBD nanopores can be obtained from the outer membrane of *E. coli* using surfactants [72] because lipid membranes and surfactants can trigger and maintain the conformational rearrangement of ClyA monomer [24, 25, 73]. We utilized this feature to successfully induce the self-reassembly of the ClyA-RBD monomer from the membrane in surfactant. The polymerized structure of ClyA has been demonstrated, and the ClyA monomer undergoes a conformational change upon membrane triggering to polymerize into a 12-mer pore structure [22, 25]. Previous studies have not solved the polymerization technology of large proteins on ClyA. We used the polymerization properties of ClyA to carry RBD to achieve polymerization. In conclusion, RBD forms a 12-mer with the polymerization of ClyA.

ClyA is a toxic protein that can punch holes in red blood cells to dissolve them. Therefore, the use of ClyA as a vaccine delivery system also presents safety concerns. However, it is encouraging that ClyA that has formed a nanopore structure on OMVs lacks hemolytic activity and loses the function of further attacking host cells [74–76]. In addition, the high hemolytic activity and consequent toxicity of the ClyA monomer can also be removed through gene mutation [77] or truncation of the C-terminus [78]. The PP used for vaccine delivery here was designed to address its safety issues. We allowed the ClyA monomer to form a complete nanopore on the bacterial membrane, thereby losing hemolytic activity. Afterwards, the bacterial membrane was removed with the help of detergents, thus ensuring that the PP lost hemolytic activity without destroying the nanostructure. In our preliminary safety experiments, we did not see obvious safety concerns, nor did we see obvious hemolytic toxicity in major organs (Fig. 5).

This article focuses on the development and use of ClyA polymerized nanopores as a novel vaccine delivery platform: SARS-CoV-2 is only one example of an application. Additionally, in contrast to animal challenge experiments, in the current evaluation of SARS-CoV-2 vaccines, neutralizing antibodies are used to reflect the level of protection conferred by the vaccine, and some innovative studies use only neutralization experiments to rapidly report vaccine technology[65, 66, 79]. At present, we hope to publish on the ClyA vaccine vector technology quickly; of course, we will also evaluate vaccine efficacy in animal models in the next step.

The keys to the success of this nanopore technique are as follows. (1) The ClyA and RBD components of the ClyA-RBD monomer separately undergo correct folding without forming inclusion bodies, and then the correctly folded oligomers are translocated to the bacterial outer membrane due to the specific secretion of ClyA to the outer membrane, thus ensuring that the correct structures of ClyA and RBD exist on the bacterial outer membrane. (2) The ClyA-RBD polymers on the bacterial outer membrane switch from membrane-triggered to surfactant-triggered [23, 25]. Therefore, stable RBD-PP can be obtained in vitro. (3) The production process of RBD-PP is subject to strict temperature and time constraints. For example, it is critical to control the process of expression in bacteria overnight at 30 °C, slow surfactant replacement at 15 °C induce polymerization into RBD-PP at 37 °C.

The RBD-PP vaccine delivery system developed here has the following advantages: (1) ClyA simulates the supporting effect of SARS-CoV-2 S2 on the RBD in S1, and the fusion of ClyA and RBD does not interfere with either protein conformation (Fig. 1B). Therefore, correct ClyA polymerization also achieves correct RBD polymerization (Fig. 1F). (2) Compared with that of monomeric RBD, the ability of regularly polymerized RBD to bind hACE2 is significantly enhanced, which illustrates the importance of RBD-PP for the efficient polymerization and exposure of RBD (Fig. 1I). (3) RBD-PP formed a stable nanostructure with a diameter of approximately 14 nm (Fig. 1F). This size is very conducive to the delivery of antigens to lymph nodes, resulting in greatly enhanced antigen uptake, processing and presentation (Fig. 2). (4) The most important point is that RBD-PP may cause this nonglycosylated modified RBD to show more spatial conformational epitopes similar to the natural RBD. (5) RBD-PP has the triple function of inducing humoral immunity and cellular immunity and activating memory T cells. Neutralizing antibodies play a major role in resisting SARS-CoV-2. It has been proven that neutralizing antibody titers above 100 can effectively resist SARS-CoV-2 infection [31, 80]. However, increasing evidence has also proven the important role of the T-cell response and immune memory in the fight against COVID-19 [81, 82]. (6) Our special preparation process not only enables ClyA to form polymerized nanostructures but also inactivates its hemolytic activity, thereby preventing the safety problems of ClyA as a vaccine carrier.

In short, the advantages of RBD-PP are due to its polymerization and its display of RBD nanostructures, which enable RBD-PP to exhibit effective immunological activation capabilities, including lymph node targeting (Fig. 2A), DC targeting (Fig. 2C), and the induction of Tfh cells (Fig. 2G) and GC B cells (Fig. 2H). Therefore, the ability of RBD-PP to induce humoral immunity (Fig. 3) and cellular immunity and to activate memory T cells (Fig. 4) was stronger than that of the RBD monomer. However, the most important point is that RBD-PP may cause this nonglycosylated modified RBD to show more spatial conformational epitopes similar to the natural RBD, which is shown by the following evidence: (1) The binding capacity of RBD-PP and hACE2 increased (Fig. 1I). (2) Four neutralizing antibodies recognized RBD-PP (Fig. 1J). (3) The ratio of neutralizing potency to binding potency increased significantly (Fig. 3H). In summary, we have developed an RBD-PP vaccine that achieves high RBD polymerization in *E. coli*, which will provide a design for an inexpensive vaccine against SARS-CoV-2.

Bacterial PPs have not previously been utilized as vaccine vectors. We developed this PP-based vaccine vector platform and conducted complete in vitro and in vivo evaluations. This also suggests that other proteins that can assemble into pores may be modified in the same way to form vaccine vector platforms for wider application. Because of the urgency of COVID-19 vaccine development, we tested the PP platform to make a vaccine against SARS-CoV-2, but different nanopores are likely to be developed in the future and applied as nanoplatforms for this and other implementations, including viral vaccines, bacterial vaccines, tumor vaccines, drug delivery, and disease diagnosis.

## Conclusions

We designed this nanopore by using the principle of ClyA porin polymerization triggered by the cell membrane and used surfactants to "pick" the ClyA-RBD nanopore from the bacterial outer membrane in this study. This was a great improvement compared to the RBD-BBV vaccine we previously reported. More importantly, the polymerized RBD displayed on RBD-PP already has some correct epitopes that can induce neutralizing antibodies. This RBD-PP promotes antigen lymph node targeting and uptake by DCs and effectively induces the production of anti-SARS-CoV-2 neutralizing antibodies, systemic cellular immune responses, and memory T cells in mice.

## Data Availability

The data used and/or analyzed to support the current study are available from the corresponding author on reasonable request.
